# Supplementary material for: Synthesis of Magnetic Ferrocene-Containing Polymer with Photothermal Effects for Rapid Degradation of Methylene Blue
Source: Polymers (Basel). 2021 Feb 13;13(4):558. doi: 10.3390/polym13040558 (PMC7918094; doi:10.3390/polym13040558)
Supplement: Supplementary file 1 [file polymers-13-00558-s001.pdf]

Synthesis of Magnetic Ferrocene-containing Polymer with Photothermal Effect for Rapid Degradation of Methylene Blue

Wenhui Zhu, Caiyun Zhang, Yali Chen and Qiliang Deng*

College of Chemical Engineering and Materials Science, Tianjin University of Science and Technology, Tianjin, 300457, P. R. China

*Corresponding author.

E-mail addresses: yhdql@tust.edu.cn (Qiliang Deng)

Photothermal Conversion Efficiency

The temperature was recorded by IR thermal camera one time per 1s for the process Deionized water of the same volume was used as a contrast. The photothermal conversion efficiency was calculated as follows:

$$\eta = \frac{hs \cdot \Delta T_{max} - Q_0}{I\ (1 - 10^{A_{808}})}$$

Where h is the heat transfer coefficient, S is the surface area of the container, ΔT_{max} is the change between the equilibrium temperature and the ambient temperature (31.7°C). Q_0 is the heat associated with the light absorbance of the solvent without polymer in the same quartz cuvette after same laser irradiation. I is the laser power (2 w·cm^{-1})，A_{808} is the absorbance of polymer at 808nm (2.429)，and η is the photothermal conversion efficiency.

hs is calculated by:

$$\tau_s = \frac{C_d m_d}{hS}$$

Where m_d is the mass of solution (1 g), C_d is the heat capacity of water (4.2 J g^{-1} k^{-1})，τ_s is calculated follows:

$$t = -\tau_s ln\theta$$

In order to get τ_s ,θ is introduced, which is calculated as follows:

$$\theta = \frac{T - T_{surr}}{T_{max} - T_{surr}}$$

Where T is the temperature of solution, T_{max} is the steady-state maximum temperature (51.1°C), T_{surr} is the initial temperature (19.3°C). Q_0 is calculated by the follow equation:

$$Q_0 = hS(T_{max} - T_{surr})$$

Based on the Fig. S1, τ_s is 329.71s. So the photothermal conversion efficiency(PCE) is 19.25%.

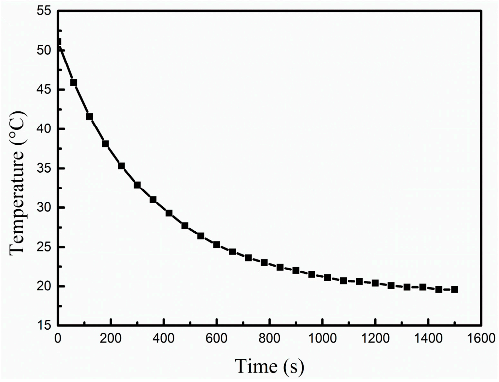

Fig.S1 The cooling period of polymer dispersion irradiated under NIR laser (808nm， 2 w cm^{-2})

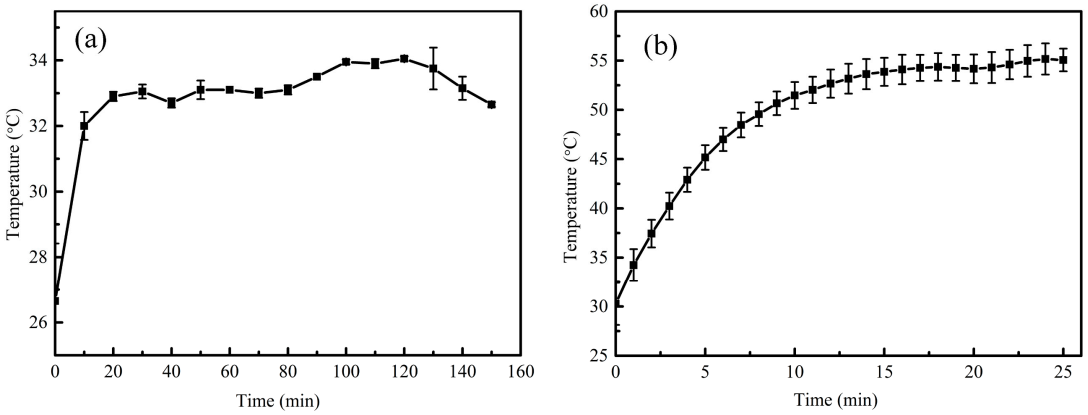

Fig S2 The temperature change under solar simulation, a power density of (a) 1 kW m^{-2} (b) 3 kW m^{-2}

Table S1. Adsorption isotherm constants of MB absorption on polymer

Model	Parameter	Value	R^2 value of best fitting
Langmuir	K_l (L g^{-1})	0.0153	0.963
	q_e (mg g^{-1})	47.941	
Freundlich	K_F ((mg g^{-1}) (mg L^{-1}) $^{1/n}$)	7.101	0.896
	n	3.378	
Temkin	K_T (L mg^{-1})	0.175	0.942
	B_T	9.847	

Table S2 Adsorption kinetic constants of MB absorption on polymer

Model	Parameter	Value	R^2 value of best fitting
Pseudo-first-order	k_1 (min^{-1})	0.00915	0.970
	q_e (mg g^{-1})	43.338	

Pseudo-second-order	k_2 (g mg^{-1} min^{-1})	0.000278	0.961
	q_e (mg g^{-1})	48.683	
Elovich	α (mg g^{-1} min^{-1})	3.682	0.912
	β (g mg^{-1})	0.133	
Intra-particle	k_3 (mg g^{-1} min$^{-1/2}$)	0.102	0.585
diffusion	C (mg g^{-1})	15.182	
